# Parenting and the Serotonin Transporter Gene (5HTTLPR), Is There an Association? A Systematic Review of the Literature

**DOI:** 10.3390/ijerph19074052

**Published:** 2022-03-29

**Authors:** Marta Landoni, Alice Dalla Muta, Sonia Di Tella, Giulia Ciuffo, Paola Di Blasio, Chiara Ionio

**Affiliations:** 1 CRIdee, Università Cattolica del Sacro Cuore, 20123 Milan, Italy; giulia.ciuffo@unicatt.it (G.C.); paola.diblasio@unicatt.it (P.D.B.); chiara.ionio@unicatt.it (C.I.); 2 Department of Psychology, Università Cattolica del Sacro Cuore, 20123 Milan, Italy; dallamuta.a@gmail.com (A.D.M.); sonia.ditella@unicatt.it (S.D.T.)

**Keywords:** parenting, 5HTTLPR, serotonin transporter, maternal sensitivity, polymorphism, gene X environment

## Abstract

The current systematic review examines whether there is an association between the genetic 5-HTTPLR polymorphism and parenting, and the mechanisms by which this association operates. The literature was searched in various databases such as PubMed, Scopus, and ScienceDirect. In line with our inclusion criteria, nine articles were eligible out of 22. Most of the studies analysed in this review found an association between 5HTTLPR and parenting. Four studies found a direct association between 5-HTTLPR and parenting with conflicting findings: two studies found that mothers carrying the short variant were more sensitive to their infants, while two studies found that parents carrying the S allele were less sensitive. In addition, several studies found strong interaction between genetic and environmental factors, such as childhood stress and disruptive child behaviour, quality of early care experiences, poor parenting environment, and quality of the environment. Only one study found an association between children’s 5HTTLPR and parenting. Parenting can be described as a highly complex construct influenced by multiple factors, including the environment, as well as parent and child characteristics. According to the studies, maternal 5-HTTLPR polymorphism is most likely to be associated with sensitive parenting.

## 1. Introduction

A large body of research has shown that parenting can have a significant effect on child development, over the short and long term [1,2]. In particular, hostile parenting could be considered a risk factor for the development of emotional, cognitive, and physical health problems in children and is also associated with higher rates of insecure attachment [3]. Several studies have specifically examined parenting, which is defined as the ability to recognise and properly respond to the signals sent by the child [4].

Despite the significant role that parental education plays in child development, few studies have been conducted to determine its predictors [2]. Indeed, parenting can be described as a highly complex construct influenced by numerous factors such as environment, education, and child characteristics [5]. In addition to these factors, genetic factors have recently been examined in the literature in relation to parenting. Mothers are often said to have a “*maternal instinct*” or a “*maternal drive,*” reflecting a widely held belief about mothers: there are innate rules, shaped by evolutionary history and embedded in DNA, that drive all mothers to respond to, nurture, and educate their children [6]. Parenting is shaped by various contextual or “external” influences, and much research has been conducted in this area. A small but growing number of studies have addressed the heritable aspects of motherhood, looking more closely at genetic variation at the molecular level to determine how DNA might structure parenting. Several genes have been considered throughout the literature. In particular, most studies have focused on the relationship between the serotoninergic system and parenting [2]. 

Although a genetic influence of 5HTTLPR on parenting behaviour in animals and other mammals is well established in the literature [7], studies in humans are still pending [2]. However, animal studies have shown that 5-HT plays a role in parental behaviour, suggesting that the serotonin system, either alone or in combination with the dopamine and oxytocin systems, may be a critical modulator of parental behaviour. 

Serotonin is a neurotransmitter involved in the regulation and control of various physiological and psychological processes [8]. In this system, the serotonin transporter (SERT or 5-HTT) plays a crucial role in regulating the availability of serotonin in the synaptic space. Therefore, much of the research has focused on the study of the gene responsible for its encoding (SLC6A4) and its polymorphic variants (5-HTTLPR) [9,10].

The 5-HTTLPR polymorphism is a functional polymorphism that involves the insertion and/or deletion of 44 pairs of nucleotide base pairs, resulting in two genetic variants: the long variant (L) and the short variant (S) [11]. The long variant is associated with higher SERT functionality and consequently lower availability of serotonin in the synaptic space [9]. The S variant, on the other hand, is associated with lower transcription of the serotonin transporter gene, resulting in increased levels of serotonin in the synaptic cleft [12].

However, studies addressing the relationship between 5-HTTLPR and parenting have reached different conclusions [2]. While the short (S) variant has been associated with higher maternal sensitivity in some studies [1,13], other researchers have found opposite results [2,14].

To explain this discrepancy, some researchers hypothesised that genetic factors might interact with environmental components through epigenetic mechanisms (gene X–environment interactions) [12,15,16].

Considering these aspects, the present review investigated the association between the genetic polymorphism 5-HTTPLR and parenting. Moreover, the study aims to draw attention to the mechanisms moderating/mediating this relationship in order to better understand the psychological transitions. To identify, assess, and synthesize the literature relevant to this topic, we focused our search on two questions:

1. Has a relationship between 5HTTLPR and parenting ever been established in humans?
2. Are there other variables involved in this relationship?

## 2. Materials and Methods

Preferred Reporting Items for Systematic Reviews and Meta-Analysis (PRISMA) guidelines were used to review quantitative research [17]. According to our objectives, we defined our questions for the literature search strategy from 2009 to 2022, the screening phase, and the extraction of the final data.

First, the screening questions were combined with inclusion and exclusion criteria [18]. Second, for quantitative studies, the acronym PICOS (Population, Intervention, Comparison, Outcome measures, and Study) was used to refine the questions and establish criteria [19].

Inclusion and exclusion criteria were: 

*Inclusion criteria*: Full-text articles that were peer reviewed, written in English, quantitative, and related to parenting skills.

*Exclusion criteria*: Studies in languages other than English; discussion papers or systematic reviews; studies conducted in animals that do not focus on parents; studies that consider only children. 

Given the paucity of articles in the literature, no criteria for measurement method (self-report and/or observational) were established.

PubMed, Scopus, and ScienceDirect were used to find articles with the following search term: 5HTTLPR AND parenting AND polymorphism in title/abstract.

The literature was reviewed to determine whether it was relevant and met the inclusion criteria. Additional articles related to the review topic were found in the reference lists of all included studies.

**Figure 1:**
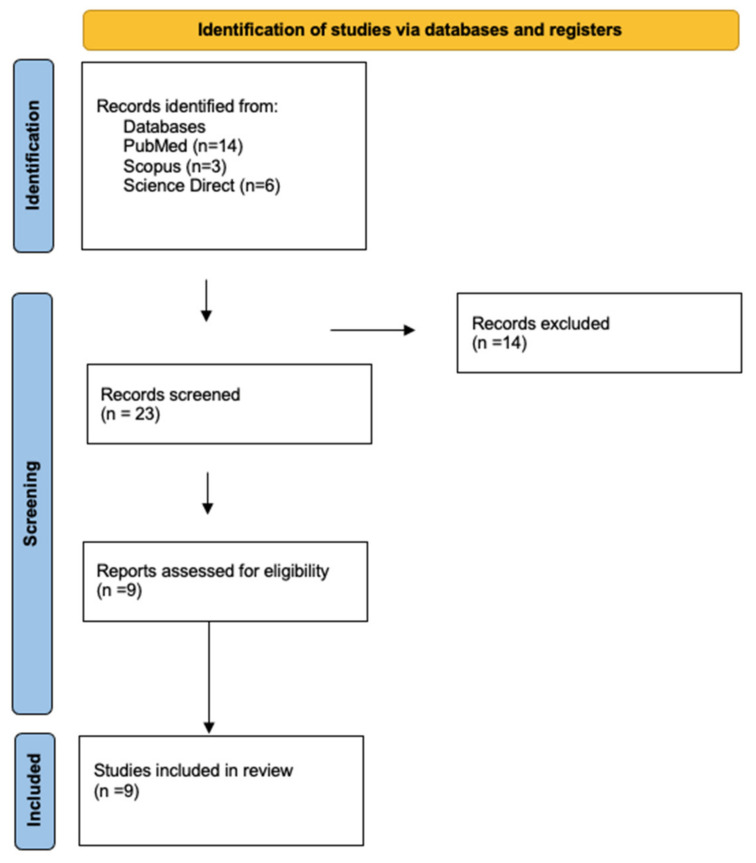
Flow diagram.

To determine if a study could be included in this review, the literature was first searched by abstract and title. The search yielded 23 articles. After screening titles and abstracts, 9 articles were included (Figure 1).

Eligibility for inclusion was assessed according to the PRISMA criteria, and the authors then discussed them to formalize agreement.

After the studies met the inclusion criteria, they were assessed using the Genetic Studies Quality Tool (Q-Genie), as shown in Table 1 [20]. This tool is used to assess and detect bias in genetic studies and is divided into nine categories: Research Design, Sample Selection, Exposure Classification, Results Classification, Sources of Bias, Presentation of Statistical Plan, Quality of Statistical Technique, Testing of Assumptions in Genetic Studies, and Interpretation of Results. 

To minimise loss of precision and reliability and to account for bias in the results, a Likert-type rating scale with seven categories anchored by “poor” and “very good” was used.

Two of the authors performed the scoring (ML and DS) and consulted in case of disagreement. 

The main findings of each study were reviewed. The magnitude of the association between parental 5-HTTLPR and maternal sensitivity was assessed using the effect size reported in the article, if available, or calculated from the data reported in the study using an online calculator (https://www.psychometrica.de/effect_size.html (accessed on 28 December 2021). Effect sizes were interpreted as follows: for Pearson’s *r* correlation, 0.1 to 0.3 as small effect, 0.3 to 0.5 as medium effect, and 0.5 and higher as strong effect; for Cohen’s *d*, 0.2 to 0.49 as small effect, 0.5 to 0.79 as medium effect, and 0.8 and higher as strong effect; and finally, Cohen (1988) gave guideline values for *η*^2^ to define small (*η*^2^ = 0.01), medium (*η*^2^ = 0.06), and large (*η*^2^ = 0.14) effects [21]. 

## 3. Results

As mentioned earlier, the analysis of the nine selected studies revealed a discrepancy in terms of methodology and characteristics (Table 2).

Regarding ethnicity, most studies examined Caucasian samples [1,2,11,12,14,23]. Only two studies addressed African [2,11] and Hispanic [2,11] samples, while three studies included Asian samples [2,16].

In terms of methodology, most studies used a combined methodology (maternal self-report and observational recordings of maternal sensitivity) [1,2,11,12,14,22,23,24]. Only one study used maternal self-assessment alone [16]. Regarding genetic analysis, all studies performed polymerase chain reaction (PCR) followed by gel electrophoresis to check amplicon size [1,2,11,12,14,16,22,23,24].

Finally, regarding the results of the studies, four of them found a direct association between 5-HTTLPR and parenthood [1,2,11,14], although the results varied, and the magnitude of the association examined using effect sizes was small. For example, Cents et al. [1] examined whether maternal 5-HTTLPR and sensitivity were associated and whether this effect was moderated by a child’s 5-HTTLPR genotype or social anxiety.

At 14 months and four years of age, results showed a consistent main effect of maternal 5-HTTLPR on maternal sensitivity: women with the S allele were more sympathetic to their children. Moreover, this association was independent of child genotype and social anxiety. These results are consistent with those of Mileva-Seitz et al. [11], who found a direct association between S/LG alleles and more sensitive parenting 6 months after birth.

In contrast, two studies found a direct association between the S allele and lower parental sensitivity [2,14]. The first study [14] showed that women with the SS genotype were less sensitive to their babies than mothers with the LL or LS genotype, even after controlling for differences in maternal education, depression, and marital strife. Morgan et al. [2] found similar results: parents with the S allele (86% of mothers) showed significantly less favourable parenting behaviours than parents with the LL-genotype.

In addition, several studies have found strong interactions between genetic and environmental factors [2,11,12,16]. Mileva-Seitz et al. [11] examined whether genotype and early caregiving experiences were related to three dimensions of maternal responsibility (maternal sensitivity, maternal behaviour away from the child, and maternal attitudes perceived attachment), and discovered highly significant GXE interactions with maternal behaviour and maternal attitudes. Specifically, mothers without S or LG alleles experienced more negative quality of early care and were more likely to distance themselves from their children. Conversely, mothers with S or LG alleles and better quality of early care reported higher scores on ratings of their perceived attachment to their baby. These findings are at odds with the observations of Morgan et al. [2], who found that infant-related stress was negatively associated with observed negative parenting in parents with the SS or SL genotype.

In addition, for both genotypes, observed child disruptive behaviour was positively associated with parental negativity, with the influence being greatest for parents with the SS or LS genotype. Sawano et al. [16] reached similar conclusions, and found an interactive effect between parenting environment and 5-HTTLPR on maternal attitudes. Specifically, a poor parenting environment (low maternal care and high paternal over-care) was negatively associated with positive attitudes toward their children in mothers with the SS genotype. In contrast, this negative effect was almost absent in carriers of the L allele. 

Different results were obtained by Baio et al. [12], who investigated whether 5-HTTLPR moderates the quality of the environmental context on maternal sensitivity. These findings revealed a GXE interaction, with women carrying the SS genotype being more responsive to the family environment than L allele bearers. Depending on whether the environment was of poor or good quality, mothers with the LL or SL genotype showed the highest and lowest levels of maternal sensitivity. Finally, two studies examined whether children’s genotype was related to maternal parenting behaviours and maternal sensitivity [22,23]. The first research [23] examined whether the effects of children’s 5-HTTLPR genotype on their mothers’ parenting behaviours were modified by their own parenting experiences. The results supported the possibility of a moderated evocative GXE association, and showed that children carrying the S allele experienced higher maternal hostility and lower maternal support, but only when the mother reported lower quality grandmotherly parenting. Belsky et al. [22] investigated whether children’s genetic profiles reduced the long- and short-term impacts of early maternal sensitivity on social-emotional and cognitive-linguistic development. Consistent with the observations of Cents et al. [1], the authors found no GXE interaction between children’s genotype and maternal sensitivity. Moreover, the children’s 5-HTTLPR did not show main effects on social-emotional and cognitive-linguistic development. 

## 4. Discussion

The present review aimed to investigate the relationship between 5HTTLPR and human parenting, highlighting the role of environment and other variables in this relationship. 

Another goal of this study was to draw attention to the processes that moderate or mediate this link in order to better understand psychological transitions and to address the objections raised in the literature.

As mentioned earlier, although the literature has demonstrated a genetic influence of 5HTTLPR on parental behaviour in animals and other mammals, very few studies have examined the link between 5HT and parental care in humans [2,24]. 

However, most of the studies included in this review found an association between 5HTTLPR and parental care. Four studies showed a direct relationship between 5-HTTLPR and parental care [1,2,11,14], although the results are contradictory and may give rise to further research and hypotheses about different mechanisms acting on this relationship. In addition, several studies found strong interactions between genetic and environmental factors [2,11,12,16], such as infant stress and disruptive infant behaviours [2], quality of early care experiences [11], poor rearing conditions, and quality of the environment [16]. Most psychological traits and behaviours are better explained by a combination of essential characteristics such as gender and socioeconomic status than by genetic markers alone, as Kagan et al. [25] recently argued, although the inclusion of both genetic and environmental factors can predict such outcomes with the greatest accuracy in certain groups.

The findings of Bakermans et al. [14] support this by showing that a lower maternal education level is more strongly associated with less empathetic parenting than genes associated with inefficient oxytocin production. It is worth noting that mothers’ empathetic interactions with their infants were observed during problem-solving tasks, which may have led to differences in empathetic parenting and explain the association with educational level. Genetic factors can be quantified more precisely than environmental influences; therefore, their effect sizes may be less comparable.

Other authors have also examined whether children’s genotype was related to maternal parenting behaviours and sensitivity [1,22,23], but only one study found an association between children’s 5HTTLPR and parenting behaviours. Specifically, children carrying the S allele experienced higher maternal hostility and lower maternal support, but only when the mother reported lower quality of parenting by the grandmother. These results can be considered consistent with twin studies showing that genetically influenced characteristics of children (e.g., temperament) trigger certain parenting measures [26].

To explain the nature of the results, there are several explanations. One of them links the 5HTTLPR polymorphism to cognitive aspects. Since the 5-HTTLPR polymorphism is associated with numerous elements of thinking function, it could have implications for parenting due to its correlation with maternal traits. Carriers of the S allele performed better on a number of tasks, including cognitive flexibility, reversal learning, attention, and inhibition [27,28,29]. 

To practice empathic parenting, parents must have cognitive flexibility and attention to effectively perceive and respond to their children’s signals [4]. For example, studies have shown that mothers with ADHD (attention deficit hyperactivity disorder) tend to exhibit behavioural characteristics of poor parents [30,31]. A reactive parenting style has also been associated with poor working memory [32]. In humans, 5-HTT binding in the putamen and midbrain is influenced by the 5-HTTLPR genotype [33]. The ventral tegmental area (VTA) in the midbrain is involved in motivation and reward as a prominent dopaminergic projection site of the mesocorticolimbic dopamine system. Based on these findings, it can be hypothesised that differences in midbrain 5-HTT function are associated with differences in dopamine signalling that influence maternal behaviour. For example, folic acid reduces 5-HTT reuptake (HT), affecting maternal behaviour in rats [34], and lesions of serotonergic neurotoxins in the median raphe, a key site for serotonin synthesis, reduce suckling and fetching in pups [34,35]. 

In addition, the 5-HTTLPR polymorphism may have a direct effect on parenting via its influence on maternal traits and neuronal and hormonal consequences. The role of oxytocin and vasopressin in determining parental behaviour in different animals seems to be significant [36,37]. 

When serotonin is activated in the hypothalamus, the paraventricular nucleus (PVN) of the hypothalamus releases both chemicals [38]. The PVN also has serotonin receptors. According to research, oxytocin and vasopressin are released through serotonin receptors [39]. When oxytocin and vasopressin systems are linked, the 5-HTTLPR may influence maternal sensitivity in parenting.

Parental genetic predisposition may be influenced by the presence or absence of stressful life conditions, with hormonal influences having greater impact in deprived contexts where social support is lacking [40]. In primates (as opposed to rodents), the scales have tipped in favour of neocortex size and function, according to Numan and Insel [41,42]. Therefore, primate parenting may be driven by cognitive rather than hormonal factors, at least in the absence of extreme conditions [41].

However, the medial preoptic area (MPOA) of the hypothalamus might still be involved in the development of complex voluntary response strategies by signalling the degree of maternal motivation to the neocortex [41]. For mothers in disadvantaged circumstances, such as those characterised by high levels of stress or marital discord, the links between serotonin and oxytocin system genes and parenting might be even more pronounced.

Finally, most studies have found an association between the S allele and parenting.

Genotypes carrying one or two S alleles have been termed “vulnerability genotypes” because they are more prone to mood disorders, especially in childhood. However, in the absence of early stress, and possibly even in the presence of early stress, the S allele may have adaptive advantages over the L allele in certain contexts. This is consistent with the emerging theory that the S allele confers increased sensitivity to environmental stimuli (Taylor 2010) and that the S allele may be viewed as a plasticity allele rather than a vulnerability allele [42].

While the S allele is often the focus of gene–environment interactions, the relationship between 5-HTTLPR genotype, early stress, and later 5-HTT function and reactivity to stress is not clear. Early stress in primates predicts increased 5-HTT expression in adulthood for all rh5-HTTLPR genotypes [43], but it is the S-bearing individuals that exhibit increased human 5-HTT expression. The L allele has a higher theoretical maximum of 5-HTT expression [44]; therefore, it may be “easier” for LL carriers to reach this maximum with even lower levels. According to Hahn and Blakely [45], the L allele may be associated with “unfavourable sensitisation to stressors in life” under certain circumstances because it is more dynamically regulated (i.e., has a higher level of transcriptional activation).

One mechanism by which the environment may influence later mothering is epigenetic effects on gene expression [46,47]. Francis, Diorio, Liu, and Meaney [48] found that mothers who lick their pups less have female offspring who lick their offspring less as adults. In addition, these female offspring exhibit decreased oestrogen alpha receptor gene expression and increased DNA methylation of its promoter region in the medial preoptic area [46]. A similar process may also affect the gene encoding brain-derived neurotrophic factor [47]. Differential gene expression via methylation is an epigenetic mechanism by which the early environment may influence long-term neuronal modulation of maternal behaviour. Since early adversity has been shown to affect methylation patterns in the human brain [49], it would be interesting to know whether the GXE effects observed in humans in this study are due to differential methylation patterns in response to early “adversity” across genotypes.

Notably, most studies found a negative correlation between the S allele and parenting attitudes [12,14,16,23,24] with the exception of three studies [1,11,12]. 

However, this discrepancy could be explained by methodological differences. For example, in this study, we recorded interactions between mothers and their 6-month-old infants at home, whereas Bakermans-Kranenburg and van IJzendoorn [14] recorded interactions between mothers and their 1–3-year-old children during problem-solving tasks in a laboratory setting. Maternal sensitivity may change over time [50], especially in mothers who exhibit depressive symptoms [51], which may be related to genetic factors. For example, sensitivity 6 months postpartum may have different genotypic associations than 12 or 36 months postpartum. In addition, mothers who are observed at home may be less stressed than mothers who are observed performing tasks in the laboratory. This may be particularly true for mothers who carry the S allele, in whom performance anxiety may lead to decreased performance or maternal sensitivity [52]. In addition, there is evidence that mother–infant interactions are different in the laboratory than at home [53]. In summary, it is possible that S-bearing mothers are more sensitive than non-S-bearing mothers in low stress situations, but less sensitive in high stress situations.

Further, several authors proposed the differential susceptibility model as an explanation.

For example, Hariri et al. [54] found that the S allele was associated with enhanced social cognition and increased amygdala sensitivity to emotional stimuli. Here, it is possible that the S allele serves as a gene of adaptability; it is more beneficial in low-risk circumstances and more challenging in difficult ones [55,56,57]. Due to their greater susceptibility to mood disorders, genotypes containing one or two S alleles have been termed “vulnerability genotypes.” Although it may increase maternal caregiving sensitivity in low-risk contexts by increasing the ability to detect environmental cues, it may also increase the risk of insensitive caregiving and hostility when the environment is more hostile.

Consistent with these observations, the expected direction of the association between 5HTTLPR and parenting is not clear [26]. However, this review has strengthened the understanding of the mechanisms involved in the relationship between genetic components and parenting.

The main limitation of current research on the influence of 5HTTLPR on parenting is the scarcity of studies and the lack of studies based on large samples. Future research could expand our knowledge of this relationship by considering other genotypes that have been identified as potential susceptibility markers (particularly MAOA, BDNF, and MR). Future studies could also consider other factors, such as ethnicity, as an important moderator in GXE studies, including genetic differential susceptibility studies [26].

## 5. Conclusions

The exploration of the influence of genetics in explaining parenthood only began around the year 2000, so it is a relatively new branch. On the one hand, this is remarkable since parenting theory can be considered the first application of evolutionary theory to human development—after Charles Darwin, but before the development of so-called evolutionary psychology.

The genotypes of the parents were identified using molecular genetic techniques. Maternal 5-HTTLPR polymorphism was associated with sensitive parenting. This study adds to the growing body of evidence showing that parenthood is a multifaceted concept. According to Swain et al. [37], parenting is a complex interplay of genes, prior parenting, current experiences, psychological state, neurobiological systems, and environmental conditions. Parenting can be better understood if we acknowledge and provide more insight into the multifactorial processes that underlie it.

Exploring possible mediators of the relationship between 5-HTTLPR and maternal sensitivity, including cognitive flexibility and attention, could provide useful insights into the underlying biological processes and provide further evidence for a link between 5-HTTLPR and parenting.

## Figures and Tables

**Table 1:** Q-Genie assessment.

	Baiao et al. 2020 [12]	Bakerman et al. 2008 [14]	Belsky et al. 2015 [22]	Cents et al. 2014 [1]	Kopala-Sibley et al. 2017 [23]	Mileva-Seitz et al. 2011 [11]	Morgan et al. 2016 [2]	Sawano et al. 2016 [16]	Sturge Apple et al. 2012 [24]
1. Rationale for study: Was a scientific rationale for chosen genes presented to avoid selective reporting of positive results?	6	6	7	6	6	6	6	5	6
2. Selection and definition of outcome of interest: Were the cases appropriately defined? Were participants appropriately sampled? Were the case/outcome assessors blinded to the genotype status?	5	6	6	5	6	5	5	5	6
3. Selection and comparability of comparison groups Were the controls appropriately defined? Were the controls sampled in a way to minimize selection bias? Was a detailed description of selection procedure (i.e., eligibility criteria, sources and methods of ascertainment, methods of matching if applicable) outlined or referenced?	N/A	N/A	N/A	N/A	N/A	N/A	N/A	N/A	N/A
4. Technical classification of the exposure Was the source (e.g., buffy coat) and method of storage for the DNA sample appropriate? Was agreement with the Hardy–Weinberg equilibrium tested in controls?	5	6	6	5	6	5	6	5	6
5. Non-technical classification of the exposure Did a blinded assessor conduct the genotyping? Was genotyping conducted in all the participants from the study simultaneously or in smaller batches?	5	6	6	5	6	5	6	5	6
6. Other sources of bias	N/A	5	7	5	6	N/A	N/A	N/A	N/A
7. Sample size and power Was the sample size appropriate? Was an a priori power analysis conducted?	5	6	6	5	6	5	6	5	6
8. A priori planning of analyses Was the analysis plan appropriate and sufficiently described?	5	6	6	5	6	6	6	5	6
9. Statistical methods and control for confounding Were important confounders appropriately controlled? Were missing data for samples and genetic variants appropriately handled? >10% missing genotype data is often unacceptable. Were the results adjusted for multiple testing to avoid false positive results?	6	6	6	5	6	6	6	5	6
10. Testing of assumptions and inferences for genetic analyses Were all assumptions concerning the genetic analysis tested?	6	6	6	6	6	6	6	5	6
11. Appropriateness of inferences drawn from results	6	6	6	6	6	6	6	5	6
Total Score	43	59	62	53	60	50	59	45	54

**Table 2:** Features of the studies.

First Author	Year	Participants	Ethnic Group	Diagnostic Instrument	Genotype	GxE	Findings	Effect Size for the Association between 5-HTTLPR and Maternal Sensitivity
Baião et al. [12]	2020	210 mothers and their preschool children. Children’s ages ranged from 40 to 77 months (M = 58.26, SD = 7.63)	Caucasian	Maternal sensitivity was measured observationally. Mother–child interaction was videotaped in a quiet room (at the family home or at the preschool) across three 5-min episodes. The mother’s ability to accurately perceive the infant’s signals was assessed using Ainsworth et al.’s (1974) 9-level maternal sensitivity scale.	SS/LL/Ls	Yes Family context	The findings revealed a gene-X–environment interaction, with short allele homozygotes proving more sensitive to the family context than long allele carriers, depending on the environmental context.	-
Bakermans-Kranenburg & van Ijzendoorn [14]	2008	159 mothers with their 2-year-old toddlers	Caucasian	During a series of problem-solving tasks, mothers’ sensitive interactions were observed. Mothers’ supportive presence, intrusiveness, and clarity of instruction were rated on 7-point scales. The Dutch Family Problems Questionnaire for marital discord. Young Adult Self-Report for maternal depression.	SS/LL/Ls	Yes Age of child, maternal education, level, depression, maternal sensitivity, or marital discord	The 5-HTTLPR SCL6A4 and OXTR rs53576 genes were found to have independent genetic effects on maternal sensitivity. Parents with the possibly less efficient variants of the serotonergic (5-HTT ss) and oxytonergic (AA/AG) system genes showed lower levels of sensitive responsiveness to their toddlers after controlling for differences in maternal education, depression, and marital discord.	partial *η*^2^ = 0.03
Belsky et al. [22]	2014	112 mothers and children	-	Child Behaviour Checklist and Teacher Report versions for total problem symptomatology. Social Skills Rating System for social competence. Mother–child interactions were videotaped during 15-min semi-structured tasks at 6, 15, 24, and 36 months.	SS/LL/Ls	Yes	There were few main effects in candidate genes, and they did not seem to interact with maternal sensitivity/insensitivity.	Cohen’s *d* = 0.11
Cents et al. [1]	2014	767 mother–child dyads. Children were assessed at 14, 36, and 48 months	Caucasian	Maternal sensitivity was repeatedly observed at the child’s age of 14 months, 36 months, and 48 months. Sensitivity was coded using the Ainsworth’s rating scales for sensitivity and cooperation and the revised Erickson rating scales for Supportive presence and Intrusiveness. Child social fearfulness was observed using the Stranger Approach episode of the Laboratory Temperament Assessment Battery at 36 months.	SS/LL/Ls	Yes Maternal age, educational level, marital status, and parity	Repeated measurement analyses revealed that maternal 5-HTTLPR has a consistent main effect on sensitivity; mothers with the S allele were more sensitive toward their children (*p* = 0.005). The 5-HTTLPR genotype of the child had no bearing on this effect. We found no evidence that the effect of 5-HTTLPR on sensitivity was moderated by child social fearfulness.	*r* = 0.17
Kopala-Sibley et al. [23]	2017	Sample 1: participants were recruited from a community sample of 405 children (208 girls) and their primary caregivers as part of a study of child temperament. At baseline, children were between 36 and 47 months of age (M = 40.72, SD = 3.51). Sample 2: participants were 476 children (251 males) and their mothers from a larger longitudinal study of 569 three-year-old children (for details, see Olino et al., 2010). The mean age of the children was 43.5 months (SD = 2.8)	European American and non-Hispanic	Sample 1: Three-bag task from which maternal support and hostility were coded. The Measure of Parenting Styles (MOPS; Parker et al., 1997) as a measure of mothers’ parenting experiences as children. Sample 2: The Teaching Tasks battery (Egeland et al., 1995), from which maternal support and hostility were rated. The Parental Bonding Inventory (PBI), a self-report measure of mothers bonding with their mothers.	SS/LL/Ls	Yes	A child with a short allele on the 5-HTTLPR gene was linked to more maternal hostility and less maternal support, but only when the mother reported poor grandmother’s parenting.	*r* = −0.01
Mileva-Seitz et al. [11]	2011	204 mothers and their children assessed to 72 months	Caucasian (90%), with 3% (n = 6) mixed ethnicity, 2% (n = 4) African, 1.5% Hispanic (n = 3), and 1% East Indian (n = 2); the rest were unknown or unspecified	At 6 months postpartum, it was recorded 30 min of non-feeding mother–infant interaction at the mothers’ homes. Maternal sensitivity was assessed using the Ainsworth maternal sensitivity scales (Ainsworth et al. 1978). The Childbearing Attitudes Questionnaire (CAQ) was used to assess mothers’ feelings and attitudes about a range of issues related to mothering and the infant. The Childhood Trauma Questionnaire (CTQ; Bernstein et al. 2003), was used to assess five types of childhood trauma: physical, emotional and sexual abuse; and emotional and physical neglect. The Parental Bonding Instrument (PBI; Parker et al. 1979), was used to assess the quality of parenting experienced during the subjects’ first 16 years of life.	S, LA, and LG	Yes	The genotype can predict differences in maternal sensitivity at 6 months postpartum, even after controlling for maternal age and parity: mothers with a S (or the functionally similar LG) allele were more sensitive than mothers without the allele during a 30-min recorded mother–infant interaction. Furthermore, highly significant gene–environment interactions in relation to maternal behaviour were found, such as mothers who lacked the S or LG alleles orienting away from their babies more frequently if they also reported poor early care quality.	Cohen’s *d* = 0.405
Morgan et al. [2]	2016	162 parents and their 6- to 9-year-old offspring. Families were sampled to include children with (n = 76) and without (n = 86) ADHD. The primary caregiver (defined as the parent who spends the most time with the child) and their child attended the laboratory in person; only the primary caregiver provided parent data. Because there was no difference between mothers and fathers in terms of positive (Z = 0.66, *p* = 0.51) and negative (Z = 0.15, *p* = 0.88) parenting, parenting data were pooled across gender.	Caucasian 62.6%, African American 8.4%, Hispanic 14.8%, Asian 7.1%, mixed 7.1%	To assess positive and negative parenting behaviours, the Dyadic Parent Child Interaction Coding System (DPICS; Eyberg, Nelson, Duke, and Boggs, 2005) was used. The task, which took approximately 20 min, required parents to play with their child in an activity of their choice. Child-related stress with the UCLA Life Stress Interview (LSI). The Parenting Stress Index–Short Form (PSI) for parenting assessment. Children’s disruptive behaviour was estimated using the number of symptoms of attention deficit hyperactivity disorder (ADHD) and oppositional defiant disorder (ODD) from the Computerised Diagnostic Interview Questionnaire for Children. IV parental depression was assessed with the Beck Depression Inventory-II. Parental ADHD was self-reported via the 18-item Adult ADHD Self-Report Scale.	SS/LL/Ls	Yes	The S allele was associated with significantly less observed positive parenting than the LL genotype. There were also significant gene–environment interactions: parental negativity was negatively associated with child-related stress in SS/SL genotype parents but not in LL genotype parents; next, observed disruptive child behaviour was positively associated with parental negativity in both genotypes, but the effect was strongest in SS/SL parents.	*r* = 0.13
Sawano et al. [16]	2016	93 mothers and their 4-month-old children	Asian	The parental bonding instrument (PBI) was used to assess the perceived quality of parental care received during the first 16 years of life. The Mother to Infant Bonding Scale (MIBS) was used to assess mothers’ affective attitude towards their own infant. The Edinburgh Postnatal Depression Scale (EPDS) was used to assess maternal symptoms of depression.	S/S l-carriers	Yes	On maternal attitude, it was discovered an interaction between the rearing environment and the 5-HTTLPR genotype. In particular, in mothers with homozygous short allele genotype, a poor rearing environment (characterised by low maternal care and high paternal overprotection) reduced a positive attitude toward one’s own infant. In long allele carriers, on the other hand, this negative effect was almost completely eliminated. Overall, our findings suggest that the 5-HTTLPR gene moderates the impact of maternal and parental behaviour on the experienced rearing environment, which is consistent with the idea that the short 5-HTTLPR allele amplifies environmental influence.	*r* = −0.09
Sturge Apple et al. [24]	2012	201 mothers and their two-year-old children	-	Mother–Child Problem Solving Task videotaped. Mother–Child Free Play/Compliance Task videotaped. Revised Conflict Tactics Scale (CTS2). Conflict and Problem-Solving Scale (CPS). Iowa Family Interaction Rating Scales (IFIRS). Empathetic Awareness Toward Children’s Needs scales from the Adult Adolescent Parenting Inventory (AAPI). Nurturance scale of the Parenting Dimensions Inventory (PDI). Computerised Diagnostic Interview Schedule IV (C DIS IV).	S/LG/LA genotypes	Yes	Mothers with one or two copies of the 5-HTTLPR S allele had a higher risk of both sensitive and harsh/punitive caregiving behaviours.	

## Data Availability

Not applicable.
